# OP34 Implications Of The New EU Regulation For Orphan Drug Health Technology Assessments In Ireland

**DOI:** 10.1017/S0266462324000965

**Published:** 2025-01-07

**Authors:** Cara Usher, Laura McCullagh, Michael Barry, Roisin Adams, Emer Fogarty, Lesley Tilson

## Abstract

**Introduction:**

Time to reimbursement has been described as a hurdle to availability of new medicines to European patients, with assessment and decision-making processes frequently quoted as taking the majority of time. In light of the upcoming Regulation (EU) 2021/2282 on health technology assessment (HTAR), the aim was to examine timelines and health technology assessment (HTA) recommendations for orphan drugs in Ireland.

**Methods:**

The study reviewed all orphan drug submissions to the National Centre for Pharmacoeconomics (NCPE) from January 2020 to December 2023 inclusive. The number of days from marketing authorization to rapid review (RR) commissioning was calculated. The RR and HTA recommendations were identified for all medicines. The timelines for the RR and HTA process were evaluated.

**Results:**

Of the 66 submissions identified, 38 percent were made prior to marketing authorization (MA), eight percent were made within 30 days of MA, and 79 percent were made 30 days post MA. RRs were completed within 32 days (mean). Full HTA was recommended in 62 percent (n=41). Price negotiations were recommended in 38 percent (50% of which have been reimbursed to date). Where a full HTA was recommended (n=41), 20 have been completed to date (price negotiations were recommended in 90%). For those 20 HTAs completed, 11 have been reimbursed to date; a decision is pending for the remainder. HTAs were completed within 200 days (mean).

**Conclusions:**

Data shows that the majority of submissions were made 30 days post MA. A pragmatic approach may have to be taken nationally to accommodate the HTAR post 2024 and those submissions that are made prior to publication of a joint clinical assessment. The majority of orphan drug HTA recommendations lead to reimbursement recommendations.

